# Miscellanies

**Published:** 1838-07-01

**Authors:** 


### MISCELLANIES.

### Results of Tapping the Head in Nineteen Cases of Hydrocephalus. By J. T. Conquest, M.D., F.L.S., &c.

*To the Editors of the Medico-Chirurg. Review.*

Gentlemen—Nearly *ten* years having elapsed since I was first induced to attempt the cure of chronic hydrocephalus, by withdrawing the fluid from the ventricles, the time seems to have arrived when the profession has a claim on me for some account of the results of these operations; and, indeed, this has become necessary in consequence of the numerous applications for information on the subject by practitioners, not only in Britain, but in many distant parts of the world. Still it is a matter involving such important considerations, that until experience has thrown much more light upon it, I do not feel justified in advancing anything beyond the mere statement of facts, such as the present position of my inquiries warrant, leaving to a future day a more methodical and full investigation of the origin, nature, and progress of this formidable disease, with its appropriate medical and surgical treatment.

The operation consists in passing a small and delicately-constructed trocar into one of the lateral ventricles, and drawing off so much of the fluid as the powers of the constitution will admit of. The most eligible spot at which the trocar can be introduced, is in the course of the frontal suture, about midway between the crista-galli process of the ethmoid bone, and the anterior fontanelle, so that the danger of wounding the corpus striatum is avoided, on the one hand, and the longitudinal sinus, on the other. The instrument usually penetrates about two inches, and in most cases the serum has been colourless, but occasionally tinged with blood. In one instance (and that was the last child operated on at *St. Bartholomew's*, only a few weeks since), a large and alarming quantity of fluid blood escaped, most likely from a branch of the meningeal artery. Sometimes, on withdrawing the trocar, the water will not flow until a probe has been passed along the canula to remove portions of cerebrum which block it up. After taking away all the fluid that can be removed consistently with safety, the head, which should always be steadily compressed by an assistant during the operation, may be strapped with adhesive plaster, that it may retain its diminished size, and that the fearful consequences of suddenly removing long-continued pressure from the brain may be averted.

I have now tapped in *nineteen* cases, and of these *ten* were living when last heard of. Several of the children, before the operation, were reduced to the most deplorable condition, having frequent convulsions, with loss of sight, emaciation, &c., but the diminution or disappearance of these symptoms has been very remarkable. In some cases the results have been triumphantly successful; in others, from the reluctance of the parents to have the operation repeated, only temporary relief has been afforded; but none of the children died either during or immediately after the operation; and those which in the subsequent list are reported as dead, survived weeks or months after the fluid was withdrawn.

All the operations were performed in the presence of many medical men, and most of them before large bodies of students at *St. Bartholomew's Hospital*, and their progress has been watched by gentlemen who have felt a deep interest in their termination, and although exclusive dependence has been placed on the withdrawal of the fluid, without the auxiliary assistance of any pharmaceutical or other remedial means, yet I consider much of the success is attributable to the kind and able superintendance of medical friends.

Having long entertained a conviction that this deplorable disease ought not to be left without something being done for its relief and cure, and not discouraged by the want of success that had followed similar attempts, and considering "anceps remedium melius quam nullum;" it was in the autumn of 1828 that I performed the *first* operation, on Catharine Seager, aged twenty months, whose head had been gradually enlarging during the previous half-year. Not more than two ounces of serum flowed, but on a probe being passed into the ventricle (by Mr. Harvey, of Islington), at the close of the day, a considerable quantity of fluid escaped per stillicidium, so that during the night it was calculated that the saturated bandages and napkins could not contain less than two pints. Only one paroxysm of convulsions followed the operation, and some symptoms of meningeal irritation which supervened were speedily subdued by leeches and cold evaporating lotions. Two years and a half subsequently I had the high gratification, in company with some friends, to see this child, when the parents left England for America; and it was not only in perfect health of body, without the slightest evidence of its having been the subject of so formidable a disease, but in full possession of its intellectual powers.

The *second* case was that of William Honey, aged eight months. The enlargement of the head had been perceptible about six weeks, and on the 20th of November, 1829, I tapped him at *St. Bartholomew's Hospital*, and withdrew twelve ounces of colourless serum from the right ventricle. On the 2nd of December twelve ounces more were withdrawn, and on the 16th an additional ten ounces and a half, making the total quantity thirty-four ounces and a half. This child was progressing most satisfactorily, when it became the subject of hooping-cough, to which intractable disease it fell a victim some months after the last operation.

The *third* operation was performed on William Wilmer, a boy now (March 1838) under a course of education in the Orphan Working School, City-road, nearly *eight years* having elapsed since twenty-four ounces of water were taken away by twice tapping him. The history of this interesting case appeared in the Lancet of September 15, 1832, by Dr. Caldwell, whose patient he was. That the account may be authenticated and impartial, the following statement is an extract from that communication: "William Wilmer, aged four months, came under my care in the month of July, 1830. His head was of an enormous size, and had been so from his birth; the forehead was large and prominent, the eyes heavy and somewhat convulsed, frequent hiccup, vomiting, &c. Several gentlemen had seen the case, and they all gave it up as hopeless. In the beginning of August Dr. Conquest performed the operation upon this child, and immediately the fluid issued forth in a stream, at first clear, and afterwards a little tinged with blood. During the remainder of the day the child continued rather weakly, but was more lively than he had ever previously been, and for some time afterwards the intensity of all the former symptoms greatly diminished.—When a month, however, had nearly elapsed, it was considered requisite to repeat the operation, and on the 3rd of September Dr. Conquest again extracted a clear liquid to the amount of twelve ounces more. The child sleeps well, eats heartily, is very lively, and is in the full enjoyment of all its mental faculties.

Signed "H. S. CALDWELL, M.D.
"Sept. 6th, 1832.

Amongst other things mentioned in the paper from which this extract is taken, is the curious fact that the head, which was enormously large at the time of the operation, remained stationary, although the size and strength of the body had gradually increased in proportion to the age of the boy, and now that nearly *eight years* have elapsed, the head, although still disproportionately large, remains at about the same dimensions.

The *fourth* case, that of Elizabeth Forster, is referred to with more than ordinary satisfaction, not only because it is the one from which the largest quantity of water was extracted (no less than *fifty-five* ounces), but more particularly because I had lost sight of the child for years, and thought it was dead, until, in September last, I received the following most gratifying communication which I transcribe entire, as it will convey all necessary information of the case:—

"Dear Sir:—Being lately on a visit in Buckinghamshire, I was enabled, through the kindness of Mr. Cowley, of Winslow, to see the child, Elizabeth Forster, residing at Little Harwood, on whom you performed the operation for hydrocephalus about *five* years since. Her countenance and general appearance are healthy; her appetite good, and her rest at night undisturbed. She has been attending a school in the village, where her progress has been equal to that of the other children; she answered questions which I addressed to her on this and other subjects with a shrewdness for which her governess says she is remarkable. The greatest circumference of the head measures 22 inches; the ossification is complete with the exception of the posterior fontanelle, and two other openings of the same size two inches apart on either side of the medial line, in the course of the coronal suture. Her mother showed a lively sense of gratitude for the benefit which she had experienced under the treatment to which you had subjected her. Your's, &c.

"Francis Cook, M.D."

It would be useless at present to detail the particulars of the other fifteen cases, as all that is important will be found in the summary given at the end of this communication, and from which it appears that of the 19 children operated upon, 10 were living, when last heard of, and 9 are dead; but it is only fair to say that as most of these children were amongst the lower classes of society, who are continually changing their residences, several have been lost sight of, and may now, very probably, be dead, although when last seen, some time subsequently to their having been operated upon, they were living.

Of course these operations have been attended with different degrees of success. Unquestionably some are cases of perfect recovery; but, in every instance, there has been a very marked diminution of suffering, and prolongation of life, and in no one case has a fatal termination been accelerated.

Dr. B. G. Babington has analysed the fluid with great care, and states its specific gravity to be 1004. It does not coagulate by heat, acids, and alcohol, and consequently does not contain albumen. Tincture of galls produces no immediate precipitate, but after standing some hours a few brown flocculi subside, proving that it contains a very little gelatine. On evaporation 1000 grains yield 10 grains of solid matter, chiefly chloride of soda, proved by precipitation with nitrate of silver. The liquid therefore contains in 100 parts—

| | |
|---|---|
| Water............ | 99. |
| Gelatine.......... | 0.1 |
| Chloride of soda.... | 0.845 |
| Other salts and loss, | 055 |
| | 100000 |

In no instance has clearly marked congenital disease been permanently benefited, and those cases have done best in which effusion manifestly resulted from inflammatory action, and in which cerebral excitement followed the operation.

The number of cases tapped, with the quantity withdrawn, will be seen in the tabular summary which concludes this brief notice of the subject.

| No. | NAMES. | Number of times operated on. | Quantity withdrawn. | Living. | Dead. |
|---|---|---|---|---|---|
| | | | Ounces. | | |
| 1 | Catharine Seager ..... | 1 | 32 | 1 | — |
| 2 | William Honey ...... | 3 | 34½ | — | 1 |
| 3 | William Wilmer..... | 2 | 24 | 1 | — |
| 4 | John Hall ............ | 5 | 48½ | — | 1 |
| 5 | Alfred Parman ....... | 4 | 45 | — | 1 |
| 6 | Mary Rayon ......... | 3 | 26 | 1 | — |
| 7 | Charles Discomb ..... | 2 | 20 | — | 1 |
| 8 | John Ward .......... | 1 | 8 | — | 1 |
| 9 | John Clauditt....... | 2 | 22 | — | 1 |
| 10 | Charles Clarke ....... | 2 | 17 | — | 1 |
| 11 | Elizabeth Forster..... | 5 | 55 | 1 | — |
| 12 | Jemima Evans........ | 1 | 7½ | — | 1 |
| 13 | Jane Brocken ........ | 1 | 13 | 1 | — |
| 14 | Eleanor Maloney ... | 1 | 9 | 1 | — |
| 15 | Frances Chiddy ....... | 4 | 33 | — | 1 |
| 16 | Thomas Norman ..... | 1 | 6 | 1 | — |
| 17 | Anne Arminio........ | 3 | 31½ | 1 | — |
| 18 | James Thompson .... | 2 | 14 | 1 | — |
| 19 | John Pratt .......... | 1 | 9 | 1 | — |
| 19 | | 44 | 455 | 10 | 9 |

I feel no ordinary pleasure in thus simply recording the progress of my investigation of this momentous and interesting subject, and shall be most happy to receive from my professional brethren any suggestions that may assist me in attempting to diminish this one source of human suffering and death.

Finsbury-square, London,
March 1st, 1838.

Knowing that Dr. Conquest is pursuing his investigations on this important subject, we are happy to afford the vehicle for invitation to contributions to the author, from such practitioners as employ the means pointed out.—*Eds.*

---

THE MEDICAL TOPOGRAPHY OF COVE. By D. H. SCOTT, M.D. (Dublin Journal, March 1838.)

Hope, which never deserts the hectic breast, has explored many a spot on the surface of this globe, in search of air and aspect that might restore the emaciated frame to flesh and plumpness—or, at all events, stave off the inevitable hour that was to sever the ties of Nature, and cut the thread of existence! How seldom these fond anticipations have been realized, the burial-grounds of Rome, Pisa, Nice, Montpellier, and fifty other health-restoring localities can mournfully tell. Still there is little doubt that many places, both on the Continent and in this country, possess great advantages over others, in cases of pulmonary affections—and happy would be the discovery of any one, within the British soil, that would supersede those places of resort in foreign countries, which few can reach, and still fewer recover in. To Hastings, Isle of Wight, Torquay, and Clifton, is now added a new asylum for pulmonary invalids on the southern shore of the Sister Isle. This is SPIKE ISLAND, at the entrance of Cove Harbour, County of Cork. Dr. Scott has given a very minute, comprehensive, and able report of its medical topography in our Dublin contemporary, and occupying fifty pages of that journal, and this paper should be perused by all those who are in the habit of being consulted as to the removal of phthisical patients from their usual domicile. It is impossible for us to give anything like an analysis of this communication, without great injury to itself and injustice to its author; yet we cannot pass it by entirely unnoticed.

The island and its village are sheltered from the winds, and open to the cheerful rays of the sun—"the site on which the town stands describing a section of a circle, whose concavity looks southward, with a rather westerly inclination—a configuration which confers on it a degree of protection, enhanced by the visits of every ray of sunshine in colder seasons." The mean annual temperature is 51.6°—the mean dew point 46°. The mean minimum temperature falls to 46.5°—and the mean weight of the atmosphere is 29.996 inches. We shall now glance at the individual months. *January* is the coldest month—its mean maximum temperature is 46.6°—mean minimum 40.4°—general medium 43.5°. *Feb.* shews an increase of one degree of temperature, chiefly by day. The North-west wind exceeds the others in prevalence. *March*, mean temperature is 45.3°. *April* exhibits an advance of three degrees of mean temperature, viz. 48.3°. In *May*, it reaches 54.7°. The easterly exceeds the westerly winds by one-third. *June* has a medium temperature of 60.2°. And in *July*, the highest range is attained, 61.9°. The south-west winds prevail in this month, and the air damp, the dew point standing very high. The thermometrical and barometrical ranges for the remainder of the year may be anticipated by what we have here stated.

As compared with Cork, the island is 2.2° warmer in Winter, whilst it is cooler by one degree in Summer. The climate is, as nearly as possible, the same as Penzance in this country; but more equable. But we can only make room for the concluding page or two of Dr. Scott's memoir, while we again strongly recommend it to the attention of the profession.

"The climate, which we have endeavoured briefly to illustrate, may be classed with those of the South West of England and of France, in its effects upon disease. Than some of these places it will perhaps be more capable of application to a larger number of cases, for from what has already been observed, the degree of moisture is less. To appreciate fully the advantages of a climate, a most essential part in the investigation of them is a right knowledge of the degree of watery vapour to the temperature. By this we learn what influence it will ever exert over one disease, or one form of the same disease more than another, and upon what fair grounds of success we can recommend this or that residence. The limits of this paper will not allow me to enter upon the morbid history of this place; I will therefore briefly state, and will reserve particulars for further consideration, those cases which will derive a benefit from a residence here. We must not forget that a climate is either preventive, remedial, or palliative, under the particular circumstances attending a case.

In dyspepsia, dependent upon an irritable state of the mucous membrane, this air is serviceable. In that form of it having its origin in a congested state of the vessels, or from nervous causes, little benefit will be derived; indeed it will decidedly disagree in general. Some uterine affections, as dysmenorrhœa, will derive advantage, the physical properties of the atmosphere contributing to relax the system, and promote secretion. Tone and vigour, have, by the combination of sea bathing, and equability of temperature, been given to the chlorotic frame, and the strumous habit so often accompanying and characterizing that disease corrected. Of rheumatism, where we find such a susceptibility to every variation of heat and moisture, and where of course, a climate subject less than another to extreme ranges, is desirable, moreover, where the hygrometric quality contributes to give a softness and mildness, remarkable in the warmer months, I can say that many cases are benefited.

The forms of asthma, which are least likely to derive advantage, are those in which the secretion from the bronchial membrane is copious; the opposite cases, for reasons sufficiently obvious, are those to which this climate has been applied with the most marked beneficial result. In affections of the larynx accompanied with diminished secretion, or that condition of the vessels productive of it, the action is salutary. In organic disease of the heart, in dilatation with or without hypertrophy, and morbid engagement of the aortic valves, many of the pectoral symptoms accompanying these are considerably alleviated, and particularly the symptomatic bronchial spasm. When bronchitis is not attended with copious secretion, depending upon a relaxed state of the mucous membrane, this air is serviceable. Some of the months will exercise this beneficial influence more than others; those for example in which the largest amount of moisture enters into the composition of the atmosphere, will not have the same effect as those in which the contrary occurs. The month of October has appeared to me to have a decidedly beneficial influence on even the chronic bronchites, with rather copious expectoration, and the same result has been observed in April and March. In that deranged state of the general health, which has been so ably described by Dr. Clarke, and which constitutes tubercular cachexy, a residence in the Winter and Spring will contribute to alter and amend the constitution. In the first symptoms of tubercular deposition, and before the frame and functions seem to be brought directly under the influence of the disease, the most marked benefit has been obtained.— When the disease of consumption has progressed farther, we cannot look for so decided a change, symptoms will be moderated and relieved, the health partially improved, and with much care and watchfulness, life prolonged. All cases of tubercular disease of the lungs, whether in their first, second, or third stages, receive a benefit, but in the two last it is too often transitory. When tubercular deposit has gone on so as to engage a large portion of the lung, when it is in its progress to softening, or when a cavity already exists, we cannot expect climate to work impossibilities. Too often are patients visiting this spot in whom, while the accents of hope fall from their lips, and prospects of lengthened days are vividly painted before them, disease has made its certain progress, and every word spoken sounds the sufferer's knell, and mocks his delusive prospects. A timely removal of a patient to any favoured climate is of paramount importance, as in the early stages of disease hygienic and therapeutic measures can be more satisfactorily applied. We witness the improvement which takes place by a change of air, in every case which presents, but the constitution in many has been brought too deeply under the disease to acquire a permanent benefit. Of the influence of the climate of Cove upon such Dr. Stokes remarks,* 'Of course, as in all cases of the kind, the good effects of the climate are seen more in the temporary improvement of the health of patients, than in their final or permanent cure. Such, however, is the penalty which all places of the sort must pay for their celebrity.' We need scarcely observe how strong therefore the necessity is, whenever a change of air is requisite, to apply its preventive or remedial influence at the earliest declaration of symptoms."

---

### Bronchotomy.

A case lately occurred in the practice of Mr. Lawrence and Dr. Johnson, which presented some interesting points of practical importance. A member of the Legislature, aged 67 years, of good constitution, was suddenly attacked with what was deemed merely a common sore throat. There was some difficulty of breathing and also of deglutition, but there was very little cough or fever, and nothing was visible on inspection of the fauces. Mr. Lawrence

* "See Dr. Stokes' highly practical and valuable work on Diseases of the Chest—a work which every man must prize who values his profession, and has the benefit of his patient at heart."

prescribed some aperient medicine and a gargle. This was on a Saturday. The symptoms, however, gradually increased, and on Monday night Dr. Johnson was called into consultation, and it was agreed that laryngitis as well as pharyngitis existed, and that active means would be necessary. As much depletion, general and local, as could be safely ventured on, in a patient of 67, was employed, while calomel, in moderate but repeated doses, were administered. As the difficulty of deglutition was much complained of, a strong solution of tartrite of antimony, grs. vj. to the ounce of water—in doses of a tea-spoonful, every four or six hours, was also given. Counter-irritation, wherever there was room beyond the bites of the leeches, was sedulously employed. Notwithstanding these, and other auxiliary measures, the malady advanced with steady and equal pace. On Thursday, it was evident that the patient was rapidly sinking, and the distress of respiration was such, that laryngotomy offered the only hope of success—and if not success, a EUTHANASIA! The patient and friends immediately assented to the operation. It was performed by Mr. Lawrence, assisted by Dr. Johnson. The incision through the integuments was in a transverse direction, between the thyroid and cricoid cartilages, and there was not a tea-spoonful of blood lost, nor did the patient complain of the least pain. The moment that an opening was made into the air-passage, a strong gust of wind burst forth, and the patient breathed with the most perfect ease. By the time that the tube was introduced and properly secured, Mr. Logan fell into a profound and tranquil sleep, breathing as easy as an infant. He was placed in bed, and slept nearly an hour. He then awoke, and made signs for something to drink. He swallowed better than before the operation. But the pulse gradually flagged, and he sunk, without the least suffering, in a few hours after bronchotomy was performed.

*Post-mortem Examination.* The body was examined by Mr. Lawrence, Dr. Johnson, and Mr. H. J. Johnson. The epiglottis was rough, rigid, and its mucous surface partially ulcerated, and what was not abraded was in a state of ramollissement. The same condition obtained in the mucous membrane lining the glottis, and the larynx, but did not extend quite so low as the wound between the thyroid and cricoid cartilages. The chief pathological feature, however, was a considerable infiltration of pus and lymph in the submucous tissue around the upper part of the larynx and also of the pharynx, by which both respiration and deglutition were greatly impeded. It is worthy of remark that on the surface of the pharynx there were one or two distinct circular pustules, exactly resembling those produced by tartrate of antimony applied to the skin. And there is every reason to believe that these were actually antimonial pustules. The difficulty of swallowing fluids in any quantity, induced the attendants to administer the antimony in the strong solution mentioned above, and as the fluid hung about the fauces, there can be little doubt that it produced pustules. This ought to be borne in mind upon future occasions, as irritation, and perhaps much mischief might ensue from the exhibition of a strong solution of tartrite of antimony. The mucous membrane of the trachea and bronchi was quite free from inflammation; and the lungs, though they seemed rather emphysematous, were otherwise sound. Thus there was nothing necessarily fatal in the state of the parts, and it may be fairly inferred that had the operation been performed 12 or 24 hours earlier, the constitution might have borne up till the parts around the larynx and pharynx had returned to a healthy condition. This gentleman certainly did not die of the operation, or of any of its consequences—neither did he die of suffocation, for he breathed with perfect freedom after the bronchotomy. We have seen the operation performed several times, and in several ways; but we certainly consider the transverse section between the thyroid and cricoid cartilages, as decidedly the most easily effected, and with least embarrassment from hæmorrhage. If disease, in any case, extend beyond this point, it will in all probability, be found to extend beyond the site of an incision into the trachea also.

---

THAMES IMPROVEMENT COMPANY.

Some seven or eight years ago, a friend of ours, Mr. Kendal, of Colchester, called on us with a plan of forming cess-pools along the banks of the Thames, for collecting the cloacal filth from the common-sewers, and rendering it available as manure for the gardens and grounds in the vicinity of the metropolis. This was one object—and that of a lucrative kind: the other, and perhaps the more important one, was, the supply of *pure water* to the inhabitants of London, by preventing the contamination of the river, from which a great portion of the water was procured. If our memory do not deceive us, Mr. Kendal proposed a terrace along the edge of the river, as a promenade for the inhabitants of both banks, and with the view of contributing to the health and pleasure of the London people. At the moment we looked upon the project as somewhat utopian; but as Mr. Kendal was a gentleman of independent fortune, we merely remarked jocosely, that the projector would probably get some soubriquet or nickname from the nature of the speculation, which, after all, might fail. It seems that the idea was abandoned by Mr. K. but we now observe that the matter has taken a tangible and practical turn, and that a company is actually formed for the purpose of carrying the speculation into existence. Dr. Granville seems to be the *primum mobile* of this great undertaking, and we sincerely wish it success —not so much on mercantile or pecuniary views, as those of Hygiene. Our readers are aware that we have always raised our voice against the abominations of the Thames water, even under all the purifications exercised on Primrose Hill, and most delighted would we be if the hundred cloacæ, opening their polluted mouths, and pouring forth the contents of their abominable stomachs into the Thames, could be stopped up, and diverted into other channels. It is true that the cloacal filth (or as Dr. Granville politely designates it—*Flemish manure)* will be spread over the market gardens, and will come back to our markets in the shapes of cauliflower, turnips, asparagus, strawberries, and various other tempting vegetables and fruits; yet still the chemical and vital processes of purification and transmutation will be very far superior to the filtering operations of the West Middlesex manufacturers. Besides, the fastidious may abstain from these cloacal products, if they please, but water is so essential to life, that we cannot evade the penalty of ingurgitating poison with every fluid we take in.

Dr. Granville has taken infinite pains during his last tour on the Continent, to collect a stupendous mass of information on the subject in question, more especially as regards the towns through which rivers pass—the precautions as to the cloacæ, or rather cess-pools—and the value of manure procured from human excretions. The Doctor calculates that between three and four pounds weight of excretions, liquid and solid, may be expected from each individual, in the course of 24 hours. This calculation appears to be corroborated by a particular establishment—the barracks at Berlin—where twelve thousand soldiers are lodged—and where a particular process is established for cloacal evacuations. It appears that barrels are the receptacles and are emptied twice a day, the manure being sold to a contractor. Admitting that there are neither women, children, nor camp-followers in the barracks to augment the cloacal deposits, we apprehend that the Berlin calculation will not apply to the Metropolis of these Isles. In the aforesaid barracks, and in the continental cess-pools generally, the fæcal matters are deposited in receptacles air and water tight, and there preserved *undiluted by the water used in the houses,* till removed by the contractor. But let it be remembered that *here,* every evacuation is instantly washed away in a deluge of water to the common-sewer. The consequence is that more perhaps than one half of the soil—and that the richest and most highly animalized—is *dissolved* as completely as opium would be, and might pass a filter without the chance of being caught. This essential difference between Continental cess-pools and British water-closets renders, in our humble opinion, all calculations that are based on the former totally inapplicable to the latter. If the company reckon on catching one fourth of the metropolitan drainings, they are probably reckoning without their host—and what they do catch will be the least valuable, because the least soluble portions of the cloacal contents.

The last, and not unimportant question is this—"can such an accumulation of human exuviæ be permitted in any one spot near the habitations of men, without injurious consequences?" Dr. Granville, in his report, p. 26, answers in the affirmative. This affirmation appears to rest on the fact that—"immediately outside of all the principal cities (on the Continent) there are large heaps of compost manure, and large reservoirs of the mixed, or semi-liquid matter removed from cess-pools; yet the neighbourhood has never been known to suffer the least inconvenience from that circumstance—*excepting the unpleasantness of the smell.*" p. 26. This last objection is got over by Dr. Granville, "inasmuch as he has acquired the knowledge of more than one process by which the offensive smell can be immediately corrected." *ib.* Dr. G. does not hint at any of these processes—but if they be any of those commonly in use for correcting mal-odorous effluvia, as chloride of lime, acid gases, &c. we very much doubt the *practicability* of applying such processes to the parallel drains proposed by the Company along the shores of the Thames.

The foregoing observations are thrown out as matters for consideration, and most sincerely do we hope that our fears are futile and our objections baseless. But we have seen enough to convince us that schemes fully as feasible as the collection of cloacal freightage, have proved impracticable in the end—or, if practicable, unproductive of profit. In respect to the Thames water itself, however filtered on Primrose Hill or elsewhere, we are still of opinion that the inhabitants of London are daily ingurgitating the very *essence* of cloacal, or, if you will—"Flemish manure," which no process short of distillation, even if that were sufficient, can separate!

---

### Bronchocele removed by Croton Oil.

This effect of croton oil rests, as yet, on a single case. A native Indian, who was goitrous, became affected with laryngitis, and Dr. Campbell (of Nepal) applied a liniment to the throat, composed of one drachm of croton oil to two drachms of olive oil. A crop of pustules came out, when the aphonia and laryngitis were removed. In fifteen days more the goitre, which, however, was small, soft, and elastic, also disappeared.—*Calcutta Transactions.*

---

### Climate and Diseases of Swan River. By Dr. Milligan.

Swan River district, the first settlement in that part of the country, and still the most important, occupies an area of about 50 miles by 30—the country being of the open forest description—the surface undulating—and the valley intersected by three rivers, influenced by the tides, and abounding in fish. The water, at some depth, is excellent—sparkles in the glass—cooks food—and washes linen well. During Summer, (our Winter) there are regular sea and land breezes—mornings and evenings pleasant—nights cool. The sky is quite Italian, without cloud, fog, or rain. In Autumn there are atmospheric perturbations, with thunder-storms that refresh the air and mitigate the heat. In Winter the winds are boisterous, with occasional heavy rains. Snow is unknown, but hail sometimes falls. The Colony was settled in 1829, and the domiciles of the settlers were, at first, of a most wretched description, but things improved rapidly till the

Winter of 1830, when want of supplies and tremendous inundations of the rivers, spread consternation and distress, followed, as usual, by diseases in the form of fever, dysentery, and scurvy, especially among the lower orders. With successive seasons the harvest became more bountiful—the flocks more numerous—and diseases less frequent. The endemics, are bronchitis, dysentery, and ophthalmia, together with a low fever which corresponds with the *gastro-enterite* of the French. The climate generally agrees with Europeans, and European animals. The annual mean temperature is from 60° to 64°. The voyage from Madras to Swan River has been performed in 25 or 30 days, and thus offers great advantages to tropical invalids. The society is good and respectable.

---

BITE OF A COBRA CAPELLA—VENESECTION CONSIDERED AS THE CURE.

This case is related by Mr. Smith, of Madras. A robust cooly was bitten by the snake, at half-past one o'clock, and he was soon insensible, breathing quick and hurried, pulse small—pupils contracted—frothing at the mouth. A ligature was placed above the bite, (belly of the biceps), and *two drachms* of the liquor ammoniæ in an *ounce* of water given immediately. Wound cauterized. In ten minutes the liq. ammoniæ was repeated, but in three quarters of an hour he was perfectly insensible, torpid and heavy—arm and hand greatly swelled. At 1 : 55 he was largely bled (more than 25 ounces was taken,) by which the circulation seemed relieved. He twice vomited a green fluid. The liq. ammoniæ was repeated, with brandy. He was now apparently sinking. Another dose of the liq. ammoniæ. He then began to revive. A fourth dose of the liq. ammoniæ, with laudanum. At 2 : 20, he was again sinking, and half-an-ounce of liq. ammoniæ, with four ounces of brandy, was given. At 2 : 30, half-an-ounce of the liq. ammoniæ with brandy. At 3 o'clock he was bled to ten or twelve ounces. We need not pursue the details, as a great number of remedies were used—including two ounces of the liquor ammoniæ, in the course of less than two hours—a most incredible quantity—seeing that the dose is from ten to twenty minims! The man recovered, but we think it very questionable whether the venesection was even the principal therapeutical agent in this case.

---

LAND SCURVY.—NUSSERABAD.

Scurvy has often broken out in besieged cities and garrisons, where food became scarce and bad, and where vegetables could not be procured; but it is rare to find it among our troops in profound peace, and where there could be little or no scarcity of provisions.

Mr. Macnab has published a paper on this subject in a recent volume of the Calcutta Transactions, which we shall briefly notice.

The senses furnished no clue to the physical agency of this intractable disease among our troops, and remedies appeared to be perfectly useless—perhaps injurious! It was attributed, and probably with justice, to some inscrutable quality of climate; though it was acknowledged that the sufferers had experienced some privations, in consequence of failure of crops the preceding year. The high prices of provisions led, of course, to frauds—perhaps to adulterations. The natives make but one enormous meal daily, and probably the digestive organs were unable to dispose of this immense cargo of rice and ghee, without detriment to the blood. For 14 years previously the troops had not suffered in this way at Nusserabad. A succession of dry seasons produce the scurvy there—even among the inhabitants. The soil is very light and sandy—and the water is saline—no jungles or exhalations. The disease commenced with the cold season—(October and November), increasing in number and severity as the cold augmented, till February, when both the weather and disease became milder. In March and April it had nearly vanished. In May it re-appeared—and in June it exhibited the most inveterate and malignant characters. Change of air was the only remedy that seemed of much advantage. "At an early stage, change of climate invariably succeeded. The transition from disease to health took place with miraculous rapidity, though for weeks before, no perceptible amendment could be obtained, under any mode of treatment."

"The following was perhaps the more regular succession of symptoms. For the first few days the patients felt some soreness in the gums, which might be seen to push up, puffy and congested, between the incisor teeth. There was languor, and perhaps a paroxysm or two of fever, or at times regular evening accessions. Day after day, the blunt projecting edges of the gums grew more prominent, became soft, and soon assumed a spongy hæmorrhagic character; the fauces, mouth, and tongue got pallid, and the breath emitted a heavy feculent odour. Now the pulse increased in frequency steadily throughout the day; irritative fever set in. Tormenting pains were developed in the muscular portions (more rarely in the tendinous) of some one or more extremities, such as the leg, arm, or thigh; seldom in more than one member, unless when the cases were of considerable aggravation. These sites became tumefied, exceedingly tense, non-elastic, and having the skin over them of a colour preternaturally dark. They were more or less diffused, sometimes occupying the whole circumference of the limb, as the leg from the knee to the ankle, which would have a shining polished appearance. Sometimes, they would only embrace a portion of the extremity, such as the calf, and then the colour would be generally darker. Vibices occasionally came out in other places, without either pain or swelling. In this state, the patient's flesh and strength wasted rapidly. The teeth, imbedded in the flabby vegetating gums, felt loose, and were unfit for the purposes of mastication. Horrid fætor now proceeded from the mouth. Despondency took active possession of the mind. There was no sleep from intense suffering, nor was there any appetite. At this period every symptom underwent a rapid advance. Extreme exhaustion would ensue, and the unfortunate subject sink eventually, and more immediately, under dropsical effusion, (a very common result,) alvine discharges, or other terminations of utter debility."

It appears, from Mr. Macnab's account, that the medical officers of Nusserabad, entirely excluded the influence of climate or locality from the etiology of this horrible complaint. To us it seems that climate or locality was almost the sole efficient cause of the malady—and that bad provisions were only the auxiliaries. The disease is curious and interesting.

---

### Dicksonian "Finish."

Our readers will remember the outrageous charges made by Dr. Dickson against his brethren, the medical officers of the Cheltenham Hospital. After several meetings of the governors and subscribers, the offer was made to this worthy to select *himself* a committee of twelve men to investigate these charges—but no! The big bully slinks into his "*imperial*" den, and dares not confront those whom he vituperated! The following editorial passage from the Cheltenham Chronicle, of May 31, would make any other man than Dr. Dickson swallow hemlock.

"THE CHELTENHAM DISPENSARY AND CASUALTY HOSPITAL.

A full meeting of the subscribers was held on Saturday, at which the medical officers and the annual committee were re-elected. Their past conduct was declared to have been highly satisfactory, and such as entitled them to unlimited confidence. We rejoice in this result, because justice is now dealt to all parties—to the accused, by removing from them every shade of imputed guilt—to the accuser, by loading him with the deserved odium of an unsuccessful attempt to impugn the characters of honourable men—and to the public, by disabusing it of any false impression that may have prevailed. He, who in a letter, dated March 29th, 1838, boasted 'I demand justice, not for myself, but for the despised and oppressed poor,' has availed himself of the only pretext that ever offered—one founded too upon an obstacle which the accused themselves speedily removed out of the way—to forsake the cause of 'the despised and oppressed poor," leaving the power of their despoilers unimpaired. Now supposing for a moment, that this person believe his charges to be true, what judgment must we form of such dastardly conduct? Here is a self-elected champion of the injured, who, after infinite boasting and bravado, suddenly leaves them to their fate, at the very time when it was in his power to make their cause triumphant. On the supposition we have just stated, such a proceeding is inconceivable; but the true reason is apparent. Falsehood is a timid thing—timid even in its daring. Yet with cautious tread it is often tempted onward by circumstances, its coward heart the while racked with disquietude. Though the evil passion—the corrupt desire—goad it forward; though the price of its iniquity lie in its path; though it be tempted by every incentive grateful to its base nature; yet does it generally fail in the hour of crisis, and fall prostrate before confronting truth."

Such is the fate which sooner or later awaits the wholesale libeller of his professional brethren!

---

Regulations and Instructions for the Naval Medical Officers. Octavo, 1835.

When the Prince Regent visited the sick-berth of the Impregnable, at Spithead, in the year 1814, while the flag of his brother, the Duke of Clarence, was flying at the mast-head, he remarked to the Editor of this Journal, (who was surgeon of the ship), that there was a great contrast between the state of things, in the time of Roderic Random, and in the time of Dr. Johnson. And so there was indeed! But even since 1814, the improvements in the naval medical department are scarcely less striking than between the days of Smollet and those of Nelson and St. Vincent. Leaving aside the immense amount of elementary knowledge which is now insisted on by the head of the department, as compared with that which existed in the revolutionary war, the improvements in the medicines, diet, and comforts of the sick sailor, are vast indeed. Instead of having the whole armamentum medicaminum squeezed into a wooden box in the cockpit, or at best under the half-deck or forecastle of a ship, dispensaries are now fitted up for the reception of the medicines and medical stores, and the chests are returned to the depôts. We observe that the pursers of all ships on foreign service are supplied with preserved meats to be issued *gratuitously,* in quantities varying from two to six ounces per man per diem, when on the sick list. But a striking feature of modern improvement in naval medicine is the establishment of "sick-messes," under the superintendance of the surgeon, partly from funds supplied by Government, when the ship is first fitted out, and afterwards by stoppages from the rations of those who are sick. The king's allowance varies from sixteen to five pounds for the first establishment of the mess, according to the size of the ship. From these stoppages, of which an accurate account is kept by purser and surgeon, the greatest comforts, and even delicacies are procured for the inhabitants of the sick-berth. This is a prodigious improvement on the olden time, when the sick or wounded sailor was dependent on the crumbs which fell from the tables of the captain and officers.

The Physician-general deserves the utmost praise for the improvements also which he has effected in the naval medical journals. The navy surgeon is obliged to make a regular nosological return—monthly at home, quarterly abroad—of the state of the sick, in a form extremely well devised, with subjoined remarks embracing a full and comprehensive account of the several diseases, the state of the weather and climate, and every other circumstance bearing on health or sickness in the ship or fleet. Independent of this an annual journal of his practice is regularly transmitted to head-quarters.

The naval surgeon on foreign stations is enjoined, as far as is practicable, to extend his observations to diseases on shore—the peculiarities of climate, the indigenous medicinal plants, to every thing that conduces to our knowledge of medical topography. The medicines are now ample in quantity, and the list contains every article in the Pharmacopœia that can be at all necessary—many more indeed than are employed by private practitioners, who are in the most extensive exercise of their profession. We have reason to believe that Sir William Burnett is exerting himself for the benefit of the naval assistant surgeons, both as respects their messes and their half-pay—subjects still of much grievance among that meritorious class of officers. We hope he will be successful.

---

### Animal Magnetism again.

We have devoted much, perhaps too much, space, to this most abominable piece of humbug. We hear that some members of our profession are from credulousness and weakness lending themselves to the propagation of the tom-foolery. We advise them to beware. The public are now gaping open-mouthed at the mountebank exhibitions that are going on, but common sense will resume its sway, and the doctors who have figured upon this occasion will find that their credulity will not be forgotten. The opinions of men of sense out of the profession even, at this moment, may be gathered from the following quotations from the Athenæum.

"The practitioners of medicine, it is true, are, for obvious reasons, more advantageously placed than others, for forming independent judgments, and, as far as that is concerned, perhaps may, *ceteris paribus*, think more soundly: but that they are professionally better logicians, more accurate appreciators of evidence, more capable of taking account of what passes in their own mind, and of separating the impressions of their senses, from the false judgments, prejudices, and inferences which become amalgamated with them at the instant of their reception, is by no means so clear."

"Not, however, to dwell upon the capabilities for reasoning exhibited by medical men, it is sufficient to appeal to the cases which they daily and almost hourly put forth with such eager confidence, announcing striking and multiplied cures, performed by certain drugs or therapeutic processes,—which drugs and processes, after a brief but extensive popularity, are abandoned as fallacious, and consigned to oblivion—and we must rest satisfied that men so circumstanced, if not pre-eminently the dupes of their own senses, are at least quite as liable to see more or less than the truth in the subjects of their observation, as others of less repute and authority."

"Besides,—we trust the credulous will forgive us for our heterodoxy,—there are, we are satisfied, many paper-headed coxcombs, many who 'will as tenderly be led by the nose as asses are,'—aye, (numbers for numbers,) fully as many in the learned professions as out of them; and we are moreover 'inclined to be of opinion,' that a fool's head is as much a fool's head when enveloped in a doctor's bonnet as when defended by a simple thrum night-cap. When, therefore, we hear of Doctor this or Doctor the other being decidedly convinced of this startling novelty, a stanch advocate for that marvellous discovery, we think the odds are rather against the discrimination of the said doctors, than in favour of the miracle."

